# Hypothalamic cannabinoid signaling: Consequences for eating behavior

**DOI:** 10.1002/prp2.1251

**Published:** 2024-08-18

**Authors:** Magen N. Lord, Emily E. Noble

**Affiliations:** ^1^ Department of Nutritional Sciences University of Georgia Athens Georgia USA

**Keywords:** CB1 receptor, endocannabinoid, energy balance, feeding, food intake, hedonic, homeostatic, leptin

## Abstract

In parallel to the legalization of cannabis for both medicinal and recreational purposes, cannabinoid use has steadily increased over the last decade in the United States. Cannabinoids, such as tetrahydrocannabinol and anandamide, bind to the central cannabinoid‐1 (CB1) receptor to impact several physiological processes relevant for body weight regulation, including appetite and energy expenditure. The hypothalamus integrates peripheral signals related to energy balance, houses several nuclei that orchestrate eating, and expresses the CB1 receptor. Herein we review literature to date concerning cannabinergic action in the hypothalamus with a specific focus on eating behaviors. We highlight hypothalamic areas wherein researchers have focused their attention, including the lateral, arcuate, paraventricular, and ventromedial hypothalamic nuclei, and interactions with the hormone leptin. This review serves as a comprehensive analysis of what is known about cannabinoid signaling in the hypothalamus, highlights gaps in the literature, and suggests future directions.

## Graphical abstract

A review of the literature found the following overarching hypotheses regarding cannabinergic signaling in the hypothalamus. High whole‐body energy status corresponds to elevated circulating leptin levels and low hypothalamic endocannabinoid tone. Low whole‐body energy status corresponds to elevated circulating ghrelin levels and high hypothalamic endocannabinoid tone. In the paraventricular hypothalamus (PVH) injections of cannabinoids produce hyperphagia, potentially via decreased glutamate and serotonin signaling and increased GABA signaling. In the lateral hypothalamus (LHA), more research is required to elucidate whether injections of cannabinoids affect intake behavior. However, evidence suggests that endocannabinoid signaling modulates the excitability of lateral hypothalamic MCH and O/H neurons. In the arcuate hypothalamus (ARH), injections of cannabinoids most likely produce hyperphagia, potentially via release of b‐End to the PVH, but the involvement of NPY is unclear. In the ventromedial hypothalamus (VMH), injections of cannabinoids most likely produce hyperphagia through an unknown mechanism. It has also been shown that endocannabinoid signaling in the VMH regulates adipose tissue maintenance and sympathetic activity. This graphical summary was created with BioRender.com.

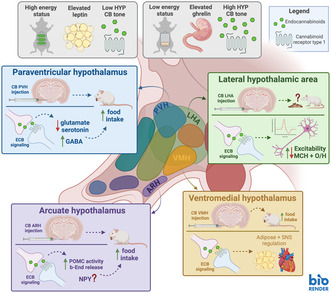

**Abbreviations**
b‐End: beta‐endorphin
CB: cannabinoid
ECB: endocannabinoid
GABA: gamma‐aminobutyric acid
HYP: hypothalamic
MCH: melanin‐concentrating hormone
NPY: neuropeptide‐Y
O/H: orexin/hypocretin
POMC: pro‐opiomelanicortin
SNS: sympathetic nervous system

## 1 INTRODUCTION

Endogenous cannabinoid compounds (endocannabinoids) are lipid‐based signaling molecules produced throughout the body, including in the central nervous system.^1^, ^2^, ^3^ Endocannabinoids exert their physiological effects, including modulating pain processes,^4^ sleep,^5^ anxiety,^6^ appetite regulation, and energy homeostasis^7^ via binding to at least two known cannabinoid receptors: CB1 and CB2 (though additional putative receptors are under investigation).^8^ Cannabinoids are so named because the first known ligands for the CB1 receptor were isolated from the *Cannabis* plant, also known as marijuana.^9^ While the phenomenon of “the munchies,” i.e., augmentation of normal food intake following marijuana use, has been described in popular culture for decades, cannabinoid research on food intake has only surfaced in the last 30 years – coinciding with the discovery of two endogenous ligands for the CB1 receptor: anandamide (AEA)^1^ and 2‐arachidonyl glycerol (2AG).^10^ Cannabinoid signaling molecules have since been clinically shown to both increase food intake and regulate overall energy balance. The hypothalamus is a brain region that orchestrates energy balance regulation,^11^ expresses CB1 receptors,^12^ and locally produces endocannabinoids.^13^ The hypothalamus has received notable attention for its contribution to cannabinoid‐induced feeding. The purpose of this review is to synthesize what is known to date about cannabinoid action in the hypothalamus with a focus on orexigenic behaviors.

CB1 receptor is a G‐protein‐coupled receptor that is ubiquitously expressed in the brain,^12^, ^14^ with the highest density of expression in the basal ganglia, hippocampal CA3 and dentate gyrus regions, and the cerebellar molecular layer.^12^ Early evidence of the role for CB1 receptor in the endogenous control of food intake comes from transgenic knockout (KO) mice. CB1 receptor KO male mice have reduced energy intake without changes in energy expenditure when compared with WT mice, and this is associated with increased lean mass, decreased fat mass, and overall reduced body weight gain starting from 5 weeks of age into adulthood.^15^ Lage and colleagues similarly report a reduction in body weight gain in whole body CB1 KO mice starting at 12 weeks of age.^16^ Interestingly, resistance to weight gain persists in whole body CB1 KO mice of both sexes following chronic high‐fat diet (HFD) feeding,^16^ despite isocaloric intake.^17^ Resistance to HFD‐induced weight gain in CB1 KO mice is coupled with blunted adiposity and lack of hyperinsulinemia compared to HFD‐fed controls, but CB1 KO mice remain susceptible to increased blood triglycerides.^17^

Evidence links the hypothalamic endocannabinoid system with energy status. For example, the endocannabinoid 2AG markedly increases in the hypothalamus of male rats after a 24‐h fast, and levels decrease below ad libitum fed controls upon refeeding.^18^ Hypothalamic AEA is unaffected by feeding, satiation, deprivation and food restriction.^18^, ^19^ However, food restriction reduces expression of the fatty acid amide hydrolase gene in the hypothalamus, the enzyme responsible for degrading AEA.^19^ This suggests that while there may not be acute changes in AEA production, long‐term food restriction may lead to elevated AEA over time, but this was not directly tested. Conversely, female rats exposed to a binge‐eating paradigm with limited intermittent access to margarine display reduced endocannabinoid tone in the hypothalamus, suggesting that binge‐eating behavior may lower endocannabinoid tone in the hypothalamus.^20^ Some evidence also suggests that hypothalamic CB1 receptor transcription may be affected by palatable food consumption.^21^ While CB1 receptor is only moderately expressed in feeding‐relevant hypothalamic nuclei,^12^ the receptor exhibits high G‐protein coupling activity in the hypothalamus in response CB1 receptor agonism.^22^ In our survey of the literature, we found several areas of focus regarding consequences of cannabinoid action in hypothalamic regions governing appetite and energy homeostasis, including the lateral, arcuate, paraventricular, and ventromedial nuclei, as well as the interplay with the adipocyte‐derived hormone leptin, which are subsequently discussed. Several endogenous, synthetic, and phyto‐cannabinoid agonists and antagonists that have been used to investigate the function of the CB1 receptor will be mentioned and abbreviated throughout the text (see Table 1). We will first highlight the established orexigenic effect of peripheral CB1 receptor agonists and the anorexigenic effect of CB1 receptor antagonist administration.

**TABLE 1:** Cannabinoid compounds that bind to the cannabinoid 1 (CB1) receptor, their origin, and binding capacity.^8^

Cannabinoid	Origin	Binding at CB1
Delta^9^‐tetrahydrocannabinol (d^9^THC)	Cannabis	Non‐selective partial agonist
CP55940	Synthetic	Non‐selective full agonist
WIN55,212‐2 mesylate (WIN)	Synthetic	Selective full agonist
AM251/AM281	Synthetic	Selective inverse agonist
HU210	Synthetic	Non‐selective full agonist
Arachidonyl‐2′‐chloroethylamide (ACEA)	Synthetic	Selective full agonist
Rimonabant/SR141716(A)	Synthetic	Selective inverse agonist/antagonist
2‐arachidonyl glycerol (2AG)	Endogenous	Non‐selective full agonist
Anandamide (AEA)	Endogenous	Non‐selective partial agonist

*Note*: “Selective” indicates selective for the CB1 receptor.

## 2 PHARMACOLOGICAL MANIPULATION OF CB1 RECEPTOR SIGNALING AND THE OREXIGENIC RESPONSE

Several studies have demonstrated central and peripheral cannabinoid administration increases food intake, and peripheral application of CB1 receptor antagonists reliably reduce feeding.^23^, ^24^, ^25^, ^26^, ^27^, ^28^, ^29^, ^30^, ^31^ In the case of agonists ACEA and CP55940, while lower doses of the drug given via intraperitoneal injection increase food intake, high doses of these CB1 receptor agonists diminish eating behavior,^29^ suggesting a bell‐shaped dose–response curve with respect to cannabinoid‐induced food intake, and these findings may be due to hypolocomotion or lethargy, which are observed at higher doses of these drugs.^30^ In addition to intraperitoneal dosing, oral delivery of THC also substantially increases food intake in sated male and female rats.^25^, ^32^ Moreover, intraperitoneal CB1 receptor blockade counters food intake in conditions when hyperphagia usually occurs, such as with hyperpalatable diets or following food restriction. For example, intraperitoneal injection of AM281 reduces acute HFD consumption in male mice,^16^ and peripheral administration of rimonabant decreases food intake in 24‐h fasted mice compared to controls.^33^ While peripheral injection of CB1 receptor agonists and antagonists have been extensively shown to increase and decrease eating behavior, respectively, the degree to which cannabinoids are acting in the central nervous system directly to modulate food intake is less clear. Indeed, lateral ventricular injection of the endocannabinoid 2AG had no impact on chow intake in free feeding male rats at any dose applied (0–160 μg icv),^18^ and lateral ventricular injection of AM251 (0–160 μg icv) to male rats had no effect on food intake nor motivated responding for food,^34^ suggesting that mediators of cannabinoid‐induced feeding are not accessible via cerebrospinal fluid (CSF) transmission in the lateral ventricle. However, WIN (3 μg icv) into the third ventricle greatly upregulates food intake in female guinea pigs compared to vehicle,^35^ and fourth ventricular injection of the CB1 agonist CP55940 is sufficient to increase palatable food intake in both male and female rats.^23^ While several factors may account for the differences observed with CSF injection of cannabinoids in the aforementioned studies, such as drug dosage and half‐life, it is also possible that the mid‐ and hindbrain injection of CB1 receptor agonists are closer to the site of action and, therefore, more accessible for cannabinoid‐mediated modulation of food intake control. Controlled studies comparing ventricular injection sites and standardized doses of CB1 receptor agonists would be revealing.

## 3 THE HYPOTHALAMUS AS A MEDIATOR OF CANNABINOID‐INDUCED EATING BEHAVIOR

While the pathway from exogenous cannabinoid administration to initiation of food intake is still under investigation, it is noteworthy that several feeding‐relevant regions of the hypothalamus are engaged by a hyperphagic dose of intraperitoneally administered CP55940 to sated male rats. This CB1 receptor agonist evoked immediate early gene expression (c‐FOS) in the lateral hypothalamus (LHA), ventromedial hypothalamus (VMH), and the paraventricular hypothalamus (PVH), as well as extra hypothalamic regions such as the nucleus accumbens shell and core.^30^ Dodd and colleagues further complemented c‐FOS gene expression data with blood‐oxygen level dependent (BOLD) imaging. Upon CP55940 administration, BOLD imaging revealed increased activity of the arcuate nucleus of the hypothalamus (ARH), and the VMH, as well as the nucleus accumbens shell and core while decreased BOLD signal was detected in the LHA and PVH.^30^ The authors note that areas showing an increase in gene expression and a decrease in BOLD signal may reflect the phenomenon of disinhibition,^30^ i.e., decreased GABA input to surrounding neurons. Endocannabinoids are produced throughout the brain and released on demand to bind CB1 receptor in retrograde fashion and acutely regulate neuronal transmission.^36^, ^37^ Thus, while it is not clear precisely where, how, nor in what order the relevant brain regions respond to peripheral cannabinoids to elevate food intake, regions with regulating capacity can be identified by conducting well‐controlled pharmacological studies.

## 4 LATERAL HYPOTHALAMUS

The LHA represents a key region modulating both homeostatic and hedonic regulation of food intake.^38^ Two populations of LHA neuropeptides are heavily involved in promoting food consumption and modulating energy expenditure: orexin/hypocretin (O/H) neurons^39^ and melanin‐concentrating hormone (MCH) neurons.^40^ Specifically, O/H neurons increase food anticipatory behavior by promoting motivation and arousal,^41^, ^42^, ^43^ and MCH signaling promotes impulsive eating^44^ and appetition^40^ as well as overall increased food intake,^45^, ^46^ which is dependent on the estrous cycle in females.^47^ Previous work has demonstrated that O/H neurons are depolarized in the immediate pursuit of food, but once food is being consumed, O/H neurons are silent and MCH neurons are activated.^48^ Interestingly, 2AG injected into the LHA has no effect on food intake alone but does increase food intake when co‐administered with the metabotropic glutamate receptor mGluR1/5 agonist.^49^ This evidence suggests an interaction between the endocannabinoid system and the O/H and MCH systems of the LHA, but glutamatergic input may also be requisite for initiating food intake, which is discussed below.

While CB1 receptors have been reported on O/H and MCH neurons,^15^ cannabinoids are also synthesized by O/H and MCH neurons. Evidence suggests these cannabinoids can act presynaptically in retrograde fashion to adjust activity in the neuron by which they were produced. This phenomenon has been termed depolarization‐induced suppression of inhibition or excitation (DSI/DSE),^37^, ^50^, ^51^, ^52^ and has been succinctly demonstrated in electrophysiological studies. Using slice electrophysiology, it was shown that direct application of the endocannabinoid AEA to MCH neurons in the perifornical LHA decreases inhibitory input from GABA neurons.^50^ Thus, endocannabinoids may bind to presynaptic CB1 receptors on GABA neurons to disinhibit MCH neurons. Furthermore, application of the CB1 agonist WIN and GABA_A_ receptor antagonist bicuculline depolarizes MCH neurons and inhibitory currents to MCH neurons are blocked by WIN; both effects are absent in the presence of AM251. In parallel, WIN also inhibits excitatory input to MCH neurons, suggesting that CB1 may also be present on presynaptic glutamate terminals that modulate MCH neuron activity.^53^ Taken together, these data suggest that cannabinoids in the LHA are adjusting fast neurotransmitter input to MCH neurons, but in vivo studies are required to understand the cumulative effect on eating behaviors.

Concerning O/H neurons, orexigenic action may be implicit in cannabinoid‐induced feeding, as antagonism of the CB1 receptor via AM251 injection into the third ventricle decreases orexin A expression^54^ and central administration of orexin A attenuates peripheral rimonabant‐induced decreases in food intake.^55^ However, in contrast to the depolarizing effect of CB1 receptor agonists in the LHA on MCH neurons, the CB1 receptor agonists WIN and AEA hyperpolarize O/H neurons and glutamate receptor antagonists attenuate the hyperpolarizing effect of WIN, suggesting that cannabinoids suppress excitatory glutamatergic input to O/H neurons.^53^, ^56^ This notion is supported by evidence showing O/H neurons synthesize 2AG in the soma and dendrites, while CB1 receptors and the enzyme responsible for the degradation of 2AG are predominantly located on presynaptic glutamate terminals when mice are fed a standard diet.^57^ Shockingly, when mice are fed a HFD, the input to O/H neurons shifts to predominantly GABAergic, as shown by triple immunolabeling,^57^ and this also the case in the *ob*/*ob* genetically obese mouse who is deficient in leptin.^56^ This is significant because CB1 receptors colocalize in these terminals, which may imply a switch from DSE to DSI,^57^ potentially removing the brakes on O/H neurons once depolarized. While sample sizes in these studies were small, overall, these observations are suggestive that consumption of a HFD and/or obesity may induce synaptic remodeling of the O/H system, potentially impacting cannabinoid‐dependent eating behavior long‐term.

Taken together, evidence suggests that cannabinoids have an important role in modulating the excitability of O/H and MCH neurons in the LHA and may even aid in the switch from O/H activity during food seeking behavior to MCH activity during consummatory behavior. However, future research is needed to understand when and how cannabinoids impact food intake control in the LHA. The balance between these neuronal populations may be disrupted with chronic HFD consumption and/or obesity, and the involvement of CB1 receptors in this phenomenon requires further investigation. Interestingly, LHA neurons may act in concert with cannabinoids in the ARH to modulate food intake. For example, hyperphagia induced by intra‐ARH orexin is attenuated by intra‐ARH injection of AM251.^58^ Orexin signaling induces release of 2AG and CB1 receptor activation in the ARH, which subsequently blunts alpha‐melanocyte‐stimulating hormone (aMSH) release in the PVH.^59^ Thus, cannabinoid signaling may be an important component of how orexin elevates food intake via modulating signaling from the ARH.

## 5 ARCUATE HYPOTHALAMUS

The ARH is a hypothalamic nucleus that is responsive to signals from the periphery regarding energy status and modulates food intake through neuropeptidergic transmission. In brief, neuropeptide Y (NPY) and Agouti‐related peptide (AgRP) neurons generally elevate food intake,^60^, ^61^ while aMSH synthesized by proopiomelanocortin (POMC) neurons and cocaine‐ and amphetamine‐related transcript (CART) neurons reduces food intake.^62^, ^63^, ^64^ Local injection of CB1 receptor agonist ACEA to the ARH increases food intake, and intra‐ARH rimonabant injection blocks peripheral ACEA increases in food intake,^29^ suggesting the net effect of cannabinoids in the ARH is hyperphagic and that ARH CB1 signaling is required for the hyperphagic effects of peripheral cannabinoid administration.

CB1 receptor is present predominantly in presynaptic GABAergic neurons in the ARH located in apposition to NPY/AgRP neurons that express diacylglycerol lipase (enzyme catalyzing 2AG synthesis),^65^ suggesting that endocannabinoid signaling in the ARH may regulate the activity of NPY/AgRP neurons. CB1 receptor mRNA and protein expression is amplified both in rats with diet‐induced obesity and, paradoxically, also in obesity resistant rats.^58^ In normal weight, overweight, and lean rats, orexin‐induced hyperphagia is attenuated by intra‐ARH injection of CB1 antagonist AM251,^58^ suggesting that ARH CB1 receptor agonism mediates orexin‐induced increases in food intake. Indeed, in rat hypothalamic explants, AEA and CP55940 increase the release of the potent orexigenic peptide NPY, and this is blocked by pretreatment with the CB1 receptor antagonist AM251.^66^ However, it is unclear if the release of NPY is via direct depolarization of NPY neurons or an intermediate mechanism.^66^ On the contrary, others have shown that bath application of ACEA in ex vivo explants reduces the activity of NPY/AgRP neurons.^29^ However, CB1 agonist WIN injected into the lateral ventricle did not affect NPY protein expression, while AM251 decreases NPY expression in the ARH.^67^ Additional in vivo evidence shows that intraperitoneal injection of the CB1 receptor antagonist rimonabant evokes similar reductions in food intake in both wild‐type and NPY‐deficient mice after 24‐h food restriction.^33^ Bringing these data together, CB1 receptor activation in the ARH increases food intake and mediates cannabinergic modulation of orexin signaling. This is likely independent of NPY signaling and may involve modulation of aMSH release in the PVH. However, more research is required to tease apart the contrary evidence presented here concerning the relevance of NPY to the cannabinergic control of food intake, including well‐designed behavioral studies to understand the impact of these interactions on eating behaviors.

In the ARH, CART and POMC are produced in the same cells and are anorexigenic.^63^, ^64^, ^68^ Acute blockade of CB1 receptor by intraperitoneal AM281 increases CART and POMC expression and reduces HFD intake.^16^ Early electrophysiological evidence suggested that CB1 receptor agonism by WIN decreases evoked excitatory currents to POMC neurons,^69^ in line with the notion that POMC neuronal activation reduces food intake. Contrary to the consensus that POMC neurons promote reductions in food intake, Koch et al.^29^ show that a hyperphagic peripheral dose of the CB1 receptor agonists ACEA or WIN induces immediate early gene expression along with increased excitatory activity in POMC neurons. However, at a high dose of ACEA showing no effect on food intake, immediate early gene expression in POMC neurons is unchanged, and this high dose of ACEA hyperpolarizes POMC neurons.^29^ The *pomc* gene transcript is a precursor for several signaling molecules, such as aMSH and beta‐endorphin (b‐End), and it has been shown that intracerebroventricular administration of WIN increases b‐End expression and immediate early gene activity in b‐End neurons.^67^ Additionally, b‐End itself injected to the lateral ventricle and directly to the hypothalamus acutely increases food intake^70^, ^71^ Rather than decreasing the release of the anorexigenic neuropeptide aMSH from POMC neurons,^72^ intra‐ARH administration of ACEA induces b‐End release from ARH projections.^29^ Moreover, peripheral injection of the mu‐opioid receptor antagonist naloxone blocks the hyperphagic effect of intra‐ARH ACEA and WIN.^29^ Hyperphagia induced by intra‐ARH injection of cannabinoid agonists is blocked by naloxone injected directly to the PVH, where ARH POMC neurons have terminals, suggesting that b‐End release to the PVH may be an essential component of the hyperphagic effect of CB1 receptor agonists in the ARH and that CB1 receptor agonists act through a mu‐opioid pathway to impact feeding. More work is necessary to fully understand the mechanism behind the selective release of peptides from multi‐peptidergic neurons, but this work suggests that cannabinoids may control the shift from release of anorexigenic to orexigenic signals in POMC neurons. Mazier and colleagues show that glutamatergic activity from POMC neurons of the ARH is dependent on 2‐AG release from PVH neurons binding at CB1. As the authors state, a decrease in energy status most likely triggers 2‐AG production and release from PVH neurons, and 2‐AG retrogradely binds to CB1, decreasing glutamatergic input to the PVH,^73^ discussed in the following section. The implications of this are that endocannabinoids may promote quick changes in excitability of appetitive signaling based on energy status.

It is clear the endocannabinoid system in the ARH modulates energy balance. Cannabinoid signaling in the ARH is required for orexin‐induced hyperphagia and may act through increasing b‐End production and decreasing aMSH release from in POMC neurons. Cannabinoids likely elevate NPY secretion from the ARH, potentially not via DSE/DSI, but rather an indirect mechanism. The impact of peripheral cannabinoid administration to directly impact ARH signaling as well as interactions with the opioid system should be more deeply considered in future studies. Endocannabinoid signaling in the PVH is discussed in more detail in the subsequent section.

## 6 PARAVENTRICULAR HYPOTHALAMUS

The paraventricular hypothalamus (PVH) houses several populations of neurosecretory cells, playing an indispensable role in energy homeostasis. The phytocannabinoid delta^9^‐THC, endocannabinoid AEA, and synthetic cannabinoid ACEA injected directly into the PVH of male rats increases regular chow intake, and this effect is blocked by CB1 receptor antagonists.^74^, ^75^, ^76^ While rimonabant alone into the PVH does not affect food intake in rats,^74^ AM251 in the PVH in ad libitum fed rats produces conflicting results,^76^, ^77^ which may be due to experimental differences (see Table 2). This pharmacological evidence points to the PVH as a site where cannabinoids are sufficient to increase eating behavior. Future studies are needed to better understand conflicting results from antagonist injections to determine if cannabinoid signaling is necessary to increase food intake in the PVH. Conflicting results may be in part explained by the mid‐range affinity both antagonists have for the mu‐opioid receptor (MOR), as AM251 has a stronger affinity for MOR than rimonabant,^78^ and MOR is expressed in the PVH.^79^ Further pharmacological and behavioral evidence discussed below reveals the interactions between the cannabinoid system and serotonin and ghrelin in the PVH.

**TABLE 2:** Differing experimental conditions in studies that injected a cannabinoid directly to the hypothalamus.

Species	Sex	CB1R agonist	Route	Energy status	Dose	Feed	Circadian	Outcome	References
Rats	Male	2AG	LHA	ad lib	1.2 μg	Rodent Laboratory Chow	Dark cycle	No effect	Sanchez‐Fuentes et al.^49^
Rats	Male	d^9^‐THC	PVH	ad lib	5 μg	Standard chow (Rat and mouse chow, Ridley AgriProducts, Australia)	Dark cycle	Hyperphagia	Verty et al.^74^
Rats	Male	ACEA	PVH	21 h fasted	0.25 μg	Standard rodent chow (LabDiet #5008)	Dark cycle	Hyperphagia	Cruz‐Martinez et al.^75^
Rats	Male	AEA	VMH	Sated	50 ng	Standard rodent chow (ARM pellets)	Mid‐light cycle	Hyperphagia	Jamshidi and Taylor^101^
Mice	x	ACEA	ARH	x	x	Standard rodent chow	Mid‐light cycle	Hyperphagia	Koch et al.^29^
Rats	Female	d^9^‐THC (l isomer)	VMH	ad lib	0.25 μg	Purina Lab Chow	x	Hyperphagia	Anderson‐Baker et al.^32^
Rats	Female	d^9^‐THC (l isomer)	LHA	ad lib	0.125, 0.25 μg	Purina Lab Chow	x	No effect	Anderson‐Baker et al.^32^
Rats	Female	d^9^‐THC (d isomer)	VMH	ad lib	0.25 μg	Purina Lab Chow	x	Reduced intake	Anderson‐Baker et al.^32^
Rats	Female	d^9^‐THC (d isomer)	LHA	ad lib	0.25 μg	Purina Lab Chow	x	Hyperphagia	Anderson‐Baker et al.^32^
Rats	Male	AEA	PVH	ad lib	100, 400 pmol	Standard rodent chow	Dark cycle	Hyperphagia	Chapman et al.^76^
Rats	Male	RIM	PVH	ad lib	0.03, 0.3, 3.0 μg	Standard chow (Rat and mouse chow, Ridley AgriProducts, Australia)	Dark cycle	No effect	Verty et al.^74^
Rats	Male	AM251	PVH	ad lib	1.6 μg	Standard rodent chow	Mid‐light cycle	Reduced intake	Soria‐Gomez et al.^77^
Rats	Male	AM251	PVH	24 h fasted	1.6 μg	Standard rodent chow	Dark cycle	Hyperphagia^a^	Soria‐Gomez et al.^77^
Rats	Male	RIM	VMH	Sated	30 μg	Standard rodent chow (ARM pellets)	Mid‐light cycle	No effect	Jamshidi and Taylor^101^
Mice	x	RIM	ARH	Fasted overnight	3 mg/kg	Standard rodent chow	Mid‐light cycle	Reduced intake	Koch et al.^29^
Rats	Male	AM251	PVH	ad lib	5, 10, 20 μg	Standard rodent chow	Dark cycle	No effect	Chapman et al.^76^

*Note*: Lowercase x indicates that the experimental detail could not be found.

^a^ Hyperphagia was not observed until 4 h post injection.

Serotonin (5′‐hydroxytryptamine; 5HT) is a monoamine neuromodulator largely released from the dorsal raphe nucleus,^80^ lesions of which induce hyperphagia.^81^ Subsequent work demonstrated the ability of 5HT signaling to reliably reduce food intake.^82^, ^83^ Evidence suggests that cannabinoids oppose the actions of 5HT in the PVH in relation to eating behavior. 5HT neurons terminate in the PVH, and 5HT acts as a satiation signal via 5HT1 and 5HT2 receptors.^84^, ^85^, ^86^ As mentioned above, ACEA, when delivered directly to the PVH, increases food intake, but when co‐administered with 5HT, food intake increases are attenuated when compared to ACEA alone^75^ suggesting a potential interaction between these two systems. In support of this, in rat PVH explants, ACEA decreases 5HT release and increases GABA release with respect to control, whereas AM251 application blocks these effects. Of note, GABAergic projections originating from the LH and terminating in the PVH have been shown to promote eating behavior, as optogenetic stimulation of these neurons initiates feeding.^87^ Like the effect of AM251, co‐administration of 5HT, a 5HT1A agonist, and a 5HT1B agonist also blocked the increase in GABA release in the PVH.^75^ These data suggest that CB1 receptor activation may be promoting GABA release in the PVH to increase food intake and that serotonin receptor signaling attenuates the cannabinergic effect of increased GABAergic signaling. The data show that 5HT signaling attenuates cannabinoid‐induced hyperphagia, likely via 5HT1A and 1B receptors in the PVH. In a freely behaving animal, this suggests that endocannabinoid release in the PVH promotes the release of GABA, promoting eating behavior, and serotonin release may promote satiation and meal termination.

Ghrelin is a potent orexigenic signal originating from the gastrointestinal tract that regulates eating behavior via the PVH.^88^ Research indicates that ghrelin systems may require endocannabinoid systems to elevate food intake. Of note, CB1 receptor KO mice do not respond to the orexigenic effects of a peripheral injection of ghrelin.^89^ Furthermore, intraperitoneal ghrelin increases hypothalamic 2AG and AEA content in wild‐type mice, which is blocked by rimonabant and absent in CB1 KO mice.^89^ Additionally, a subthreshold subcutaneous injection of rimonabant that has no effect on food intake alone prevents intra‐PVH ghrelin‐induced hyperphagia.^90^ These data taken together suggest that ghrelin signaling in the PVH works in concert with the cannabinoid system to promote food intake. As it has been iterated several times in this review, CB1 receptor antagonists generally decrease food intake or show no effect. Evidence from Soria‐Gomez and colleagues contradicts this working knowledge of CB1 receptor antagonists, as intra‐PVH AM251 injected at the end of a 24‐h fast *increases* fasting‐induced hyperphagia.^77^ Specifically, AM251 given in conjunction with ghrelin directly into the PVH *potentiates* the hyperphagic effect of ghrelin in free‐fed rats, but not until 4 h post treatment.^77^ The possibility that the acute effect of AM251 is expired after 4 h, however, should not be excluded. Much of the evidence points to an inverse relationship between circulating ghrelin and hypothalamic endocannabinoids, and CB1 receptor being required for ghrelin‐induced increases in food intake.

Cannabinoid action in the PVH likely has an overall potentiating effect on food intake working in conjunction with ghrelin and in opposition to 5HT. Ghrelin likely acts to increase food intake partially by elevating hypothalamic endocannabinoid content, and cannabinoids may promote food intake in the PVH via increasing GABA release and suppressing 5HT release. Work from Rorato and colleagues shows that acute and prolonged (1 week) treatment with rimonabant increases CB1 receptor mRNA in the PVH,^91^ but there is a notable lack of evidence of the location of CB1 receptors in the diverse PVH region.^15^ Future study should shed light on production and local signaling of endocannabinoids as well as receptor localization in this region to better understand the perplexing behavioral data gathered from these few studies. Relevant to overall energy balance, AEA administration to the PVH increases the respiratory quotient,^76^ indicating potential changes in energy expenditure. Interestingly, when the CB1 receptor is knocked out of Sim1 neurons (expressed by most neurons of the PVH), no changes in energy expenditure could be detected until mice were maintained on an HFD when animals lacking Sim1 neuronal CB1 receptor showed increased energy expenditure and adrenergic receptor gene expression,^92^ suggesting that the CB1 receptor in Sim1 neurons is responsive to the metabolic effects of an HFD.

## 7 VENTROMEDIAL HYPOTHALAMUS

Like other regions discussed, the ventromedial hypothalamus (VMH) has moderate expression of CB1 receptor in both GABAergic and glutamatergic synapses.^93^ Steroidogenic factor 1 (SF1), a transcription factor exclusively produced in the VMH^94^ that has been implicated in energy balance regulation via leptin^95^ and glucose^96^ sensing, is necessary for the expression of CB1 receptor in the VMH.^97^ Kim et al.^97^ found that SF1 can directly stimulate CB1 receptor transcription activity via two potential binding sites in the promoter region of the gene, and agonism of the CB1 receptor by WIN decreases the firing rate of SF1 neurons. This suggests that SF1 is regulating the expression of CB1 receptor, which, in turn, regulates the activity of SF1 neurons.^97^ Two groups have contributed data to the field examining the metabolism of mice wherein the CB1 receptor has been specifically knocked out of SF1‐positive neurons. Neither group found differences in body weight or lean mass in male SF1‐CB1‐KO mice on standard chow.^98^, ^99^ However, Cardinal et al. found reduced fat mass and modest improvements in glucose and insulin tolerance, while Castorena et al., found no differences in fat mass nor plasma insulin.^98^, ^99^ Cardinal et al.,^98^ further examined markers of sympathetic activity due to exhibition of a reduced respiratory quotient in male SF1‐CB1‐KO mice indicating a preference for fat as energy substrate compared to carbohydrates. Male SF1‐CB1‐KO mice on chow showed increased adrenergic receptor expression and increased phosphorylated hormone sensitive lipase expression in white adipose tissue relative to WT mice.^98^ Interestingly, when SF1‐CB1‐KO mice were put on a HFD for 8 weeks, these measures inverted, i.e., modest deterioration of glucose tolerance, increased respiratory quotient, decreased adrenergic receptor expression, and decreased phosphorylated hormone sensitive lipase expression in white adipose tissue.^98^ SF1‐CB1‐KO mice further displayed increased total HFD intake, total body weight, and fat mass on HFD, with no differences in lean mass nor insulin tolerance.^98^ Castorena et al.,^99^ contradict this evidence showing male SF1‐CB1‐KO mice measures of weight, body composition, nor food intake do not change when placed on a HFD. However, SF1‐CB1‐KO males did display decreased plasma glucagon, decreased hepatic glucose production, and increased glycogen synthase,^99^ in line with decreased sympathetic activity when placed on HFD. Female SF1‐CB1‐KO mice on standard show or a HFD did not show differences in weight, lean or fat mass, plasma insulin, glucose or insulin tolerance.^99^ A separate study by Cardinal et al.^100^ showed virally‐mediated CB1 knockdown (60% reduction) specifically in the VMH reduces weight gain compared to control littermates driven by increased energy expenditure in both phases of the diurnal cycle, while total locomotor activity and food intake are not different from wild‐type mice. They replicate the elevation in adrenergic receptor mentioned above and further show elevated uncoupling protein‐1 mRNA gene expression.^100^ Inconsistencies between studies may be due to differences in KO model generation, housing conditions, or diet composition, as noted by the authors.^99^ Overall, these data demonstrate that CB1 receptor in the VMH, specifically in SF1 neurons, regulate sympathetic activity and contribute to adipose tissue maintenance, and these functions are dysregulated in the presence of HFD.

There is limited pharmacological evidence investigating how the body responds to exogenous cannabinoids in the VMH. Early investigations by Jamshidi and Taylor showed that AEA injected into the VMH of sated male rats increases standard food intake with a bell‐shaped dose response.^101^ Additionally, intraperitoneal injection of CB1 agonists (methanadamide and ACEA) to 24 h fasted male and female mice augments standard chow intake in WT mice, but this effect is absent in SF1‐KO mice,^97^ suggesting that CB1 receptor and/or SF1 is required for the hyperphagic effects of peripheral CB1 agonists. Similarly, AM251 blunted refeeding in WT mice, but not in SF1‐KO mice.^97^ Together these data suggest that SF1 in the VMH is required for cannabinoid‐induced hyperphagia and given that SF1 is required for CB1 receptor expression it is possible that these drugs work directly on CB1 receptors in the VMH, but this possibility has not yet been directly tested.

## 8 HYPOTHALAMIC LEPTIN SIGNALING AND THE ENDOCANNABINOID SYSTEM

Leptin is an adipocyte‐derived hormone that increases proportionally to body fat, and leptin is one of the hormones that relays to the hypothalamus information regarding the body's energy status.^102^ There is ample evidence for an interaction between hypothalamic leptin signaling and CB1 receptor signaling in the regulation of energy balance. CB1 receptor null mice have decreased plasma leptin, coinciding with decreased fat mass,^15^ and this is true even for CB1 receptor null mice that are fed a HFD.^16^ Furthermore, the hypophagic effect of peripheral leptin injection is abolished in mice with hypothalamic specific CB1 receptor knock out,^100^ but the mechanism remains unclear. In line, exogenous leptin given to diet‐induced obese male rats in combination with rimonabant results in elevated weight loss compared to either treatment alone and vehicle.^103^ Interestingly, intravenous delivery of leptin reduces hypothalamic levels of 2AG and AEA in male rats.^33^ Further, animals lacking the leptin receptor display increased hypothalamic levels of endocannabinoids, and mice lacking leptin itself also present with increased 2AG in the hypothalamus, which returns to control levels upon leptin administration.^33^ Conversely, AEA levels in the *ob*/*ob* mouse, which lacks the ability to produce functional leptin, are not different from control mice, but leptin administration significantly decreases AEA below control values in these animals.^33^ Taken together, leptin signaling reduces hypothalamic endocannabinoid levels and endocannabinoid signaling may be necessary for the normal effects leptin.

There are several lines of evidence examining CB1 receptor and leptin receptor expression in the hypothalamus,^104^ specifically in the VMH and PVH. In the PVH of male Zucker rats that lack the leptin receptor, no differences could be detected in CB1 receptor expression in lean or obese rats regardless of prandial state.^105^ However, authors do note changes in CB1 receptor expression in the VMH of obese leptin receptor deficient rats in that ad libitum fed rats have elevated CB1 receptor expression, while fasted rats have diminished CB1 expression compared to lean controls.^105^ Specific to the VMH, deletion of CB1 receptor from SF1‐positive neurons has direct consequences on leptin signaling in the VMH.^98^ While WT and SF1‐CB1‐KO have indistinguishable leptin receptor expression, SF1‐CB1‐KO mice are more sensitive to leptin as shown by a 20% decrease in 24 h food intake and greater phosphorylated‐STAT3 expression in the VMH.^98^ These changes were coupled with elevated adrenergic receptor and hormone sensitive lipase gene expression and a reduced respiratory quotient.^98^ When SF1‐CB1‐KO mice were placed on a HFD for only 2 weeks, the sensitivity SF1‐CB1‐KO mice displayed on chow was abolished with markedly decreased phosphorylated‐STAT3 expression.^98^ These data suggest that CB1 receptor in the VMH reduces leptin receptor sensitivity in the VMH with consequent susceptibility to weight gain.

## 9 CONCLUSIONS

Endocannabinoid signaling plays an essential role in eating behaviors coordinated by the hypothalamus. Evidence reviewed here suggests that cannabinoid signaling is critical to the integration of peripheral signals, as lack of the CB1 receptor negates the hypo‐ and hyperphagic effects of leptin^100^ and ghrelin,^89^ respectively. Additionally, hypothalamic endocannabinoid tone may be dependent on energy status. Fasting and feeding have inverse effects on endocannabinoid production,^18^ but there is little data to attest to how these energy states affect CB1 receptor expression^105^ or membrane localization, and sex differences in endocannabinoid regulation may be present^19^ but are understudied. There is consensus that CB1 KO mice weigh less, but whether this is due to caloric deficit or increased energy expenditure is still debated. Knocking down the CB1 receptor in the VMH increases energy expenditure in both phases of the diurnal cycle, generating supporting evidence for the latter hypothesis. While CB1 receptor is expressed in SF1 neurons of the VMH, CB1 receptor expression in the PVH has been shown to colocalize with CRH^15^ and is expressed in Sim1 neurons,^92^ and further characterization of CB1 expression on the various neurons of the PVH controlling endocrine function may shed light on cannabinoid interactions with energy expenditure.

Strong evidence suggests that CB1 receptor signaling in the PVH is critical for CB1‐mediated orexigenic effects, but more data are needed (site‐specific antagonist injections/receptor knockdown) to pinpoint the roles of other hypothalamic regions in orchestrating cannabinoid‐induced eating behavior. Indeed, there are many outstanding questions regarding CB1 signaling in the hypothalamus. Further research is needed to investigate how cannabinoids may be increasing GABA release in the PVH,^75^ as this data point is incongruent with the hypothesis of disinhibition. In the arcuate nucleus, there are several lines of conflicting evidence attempting to elucidate how cannabinoids are interacting with NPY/AgRP neurons. It stands to reason that the cannabinoid system may regulate NPY neuron activity, but more in‐depth analysis of this potential interaction is needed to make sense of the existing literature. Other outstanding questions in the arcuate nucleus include the potential role of cannabinoids in regulating or participating in the selective release of peptides from multipeptidergic neurons, such as POMC neurons. Solving one such mystery of this nature may additionally give us clues into how cannabinoids may be regulating other multipeptidergic neurons involved in food intake control, such as MCH neurons. Finally, evidence suggests that endocannabinoid signaling is modulating the excitability of lateral hypothalamic MCH and O/H neurons, but the effect on intake behavior, if any, remains to be determined.

Regarding the relationship between HFD feeding and the endocannabinoid system, the evidence indicates that lack of cannabinoid signaling is protective against the negative metabolic effects of a HFD,^17^ and antagonism of the CB1 receptor reduces HFD consumption.^16^ However, the long‐term effects of HFD feeding on the endocannabinoid system are unclear. HFD feeding may alter leptin sensitivity in the VMH through a CB1 receptor‐mediated mechanism,^98^ but the endogenous relevance of the CB1 receptor to VMH‐controlled energy balance is still under investigation.^98^, ^99^ Most interestingly, limited evidence demonstrates that chronic HFD consumption potentially induces synaptic remodeling in O/H neurons, increasing the density of O/H innervation to target regions.^57^ In the acquisition of a meal, it has been shown that O/H neurons are active until meal initiation, and, subsequently, previously silent MCH neurons are depolarized during meal consumption.^48^ Given the impact of HFD feeding on O/H neurons, it may be suggested that HFD feeding perturbs this balance between lateral hypothalamic neurons and should be a topic of further investigation.

The scientific debate these studies conjure up can sometimes be simply attributed to differences in experimental design. Experimental conditions likely contribute to differences noted in pharmacological manipulations of the cannabinoid system. In particular, ventricular injections have yielded highly inconsistent results, and this may be due to dosing and distance from injection to the site of action. Controlled study comparing lateral, third, and fourth ventricular injection sites with standardized dosing of CB1 receptor ligands would aid in alleviating the existing discrepancies in the literature. Concerning intrahypothalamic injections of cannabinoids, the differing experimental conditions and resulting behavior are noted in Table 2.

While the intersection of cannabinoid and neuroscience research has made great strides in the past few decades, the field has many questions yet to be answered. There is still a dearth of research focusing on the endocannabinoid system and cannabinoid‐induced eating in female subjects. To have a complete picture of how the endocannabinoid system is orchestrating energy intake, female subjects must be included in all future endeavors.

## AUTHOR CONTRIBUTIONS

MNL wrote the original manuscript and EEN and MNL edited the manuscript.

## FUNDING INFORMATION

This manuscript was supported by DK118000 (EEN).

## CONFLICT OF INTEREST STATEMENT

The authors have no conflicts to disclose.

## ETHICS STATEMENT

Not Applicable.

## Data Availability

Data sharing is not applicable to this article as no new data were created or analyzed in this study.
